# Microwave ablation of breast pseudoaneurysm

**DOI:** 10.12669/pjms.38.1.4930

**Published:** 2022

**Authors:** Zhenyu Cai, Ran Ning, Wenwu Dong, Yiqing Zhang

**Affiliations:** 1 Zhenyu Cai, Department of Ultrasound, Hangzhou Hospital of Zhejiang Medical and Health Group, Hangzhou 310022, China; 2 Ran Ning, Department of Ultrasound, Hangzhou Hospital of Zhejiang Medical and Health Group, Hangzhou 310022, China; 3 Wenwu Dong, Department of Ultrasound, Hangzhou Hospital of Zhejiang Medical and Health Group, Hangzhou 310022, China; 4 Yiqing Zhang Diagnosis & Treatment Center of Ultrasound Medicine, Zhejiang Xiaoshan Hospital, Hangzhou 311201, China

**Keywords:** Breast, pseudoaneurysm, microwave ablation, experience

## Abstract

Breast pseudoaneurysm is a very rare complication. In this study, we report a patient with huge breast pseudoaneurysm after ultrasound-guided vacuum-assisted biopsy (UGVAB) of breast nodules. In treatment, we used microwave ablation to treat the pseudoaneurysm, and then used UGVAB again to eliminate the complicated hematoma. The patients obtained good therapeutic effect. From this case, we experience that, before the interventional operations for breast nodules, the systematic ultrasound examination should be performed. In the needle entering channel, the obvious blood vessels should be avoided to reduce the unnecessary vascular injury. When the pseudoaneurysm occurs, the patient’s condition, pseudoaneurysm situation and hematoma size should be comprehensively considered, combined with the multidisciplinary consultation, for selecting the best treatment strategy.

## INTRODUCTION

Pseudoaneurysm often occurs in the main and middle arteries. The most common sites of traumatic pseudoaneurysm are the femoral artery, carotid artery, ulnar artery, etc..^1^ Pseudoaneurysm is very rare in the small arteries such as superficial thoracic artery. Ultrasound-guided vacuum-assisted biopsy (UGVAB) is widely used in breast tumor biopsy and minimally invasive resection of benign breast tumor. The most common complications of UGVAB are the hematoma and infection.^2^ The superficial thoracic artery, a blood-supply vessel of breasts, has the small diameter, and its complications are rare. There are occasional reports of breast pseudoaneurysm.^3^,^4^ We speculate that, the main influencing factor of pseudoaneurysm treatment is the tumor diameter. In our hospital, there was a patient with huge breast pseudoaneurysm after UGVAB. In therapy, we used microwave ablation to treat the pseudoaneurysm, and then used UGVAB again to eliminate the complicated hematoma. Herein, with the informed consent of the patient, we presented a summary report.

## CASE PRESENTATION

**Fig.1:**
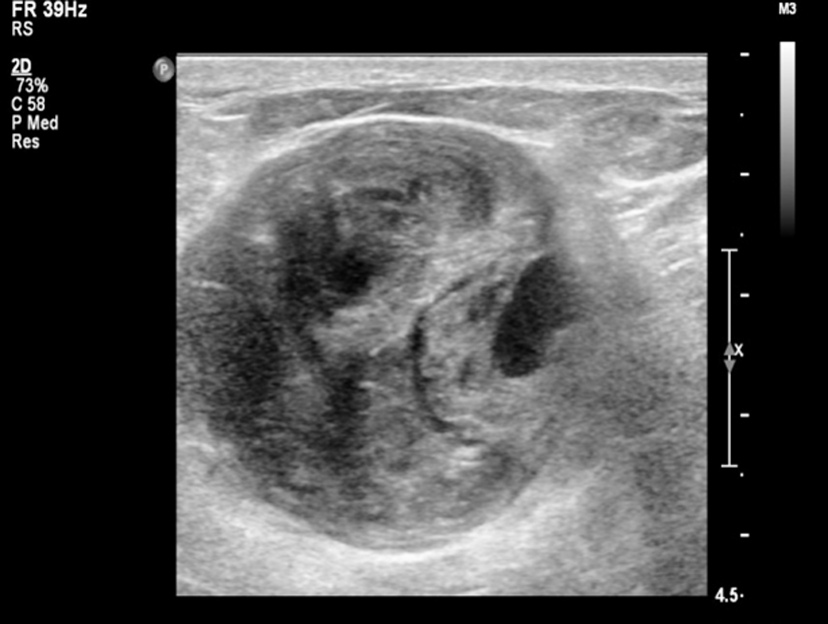
Conventional ultrasound of right breast pseudoaneurysm combined with hematoma.

**Fig.2:**
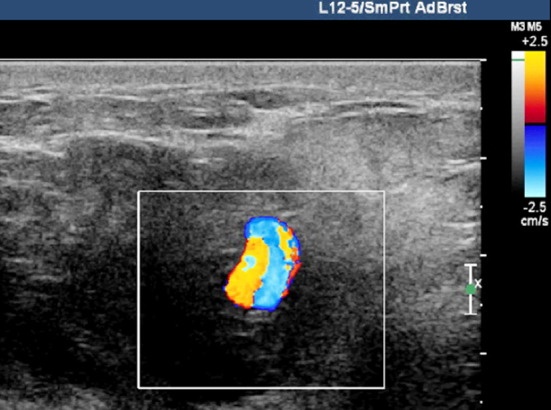
Color Doppler ultrasound of right breast pseudoaneurysm combined with hematoma.

A 48 year-old woman was found with multiple nodules in her breasts during the routine physical examination. The patient came to our hospital, and asked the minimally invasive treatment of two larger nodules. Under the ultrasound guidance, the UGVAB was performed using the 9-guage rotary cutting needle. The histopathology showed the breast sclerosing adenosis combined with reactive lymph node hyperplasia in the right-side mass and breast fibroadenoma combined with local acute and chronic inflammatory cell infiltration and micro abscess in the left-side mass. On the third day after the surgery, the patient came back to the hospital. She complained that, after the removing the bandage at postoperative 48 hour, there was a mass in the right breast. The mass then became larger and larger, and the pain became more and more serious. When she arrived at the hospital, she was difficult to lift the right hand up. The conventional ultrasound examination showed an uneven hypoechoic mass (about 6.7 cm * 3.5 cm * 5.3 cm) in the right upper breast quadrant, with a pulsatile dark area (about 1.1 cm * 0.7 cm * 0.5 cm) at the inner edge (Fig.1). The color Doppler ultrasound showed red-blue alternating blood flow signals, with biphasic arterial spectrum (Fig.2). This suggested that the pseudoaneurysm accompanied by huge hematoma was formed.

**Fig.3:**
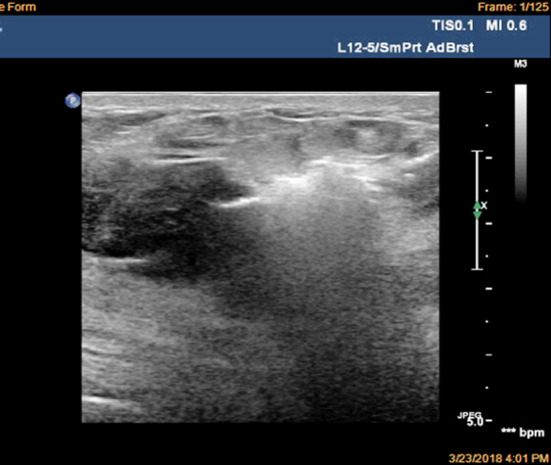
Microwave ablation of breast pseudoaneurysm.

**Fig.4:**
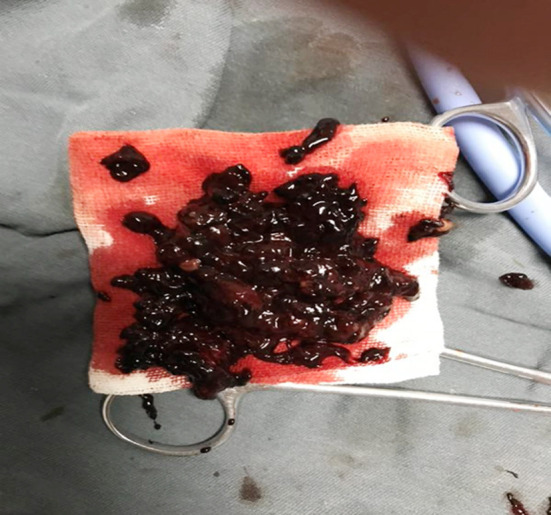
Ultrasound-guided vacuum-assisted biopsy of hematoma.

In the treatment of pseudoaneurysm, the commonly used methods included the ultrasound-guided local directional compression, percutaneous prothrombin injection,^5^,^6^ surgical ligation of blood vessels and hematoma resection.^7^,^8^ For this patient, after diagnosis the ultrasound-guided local directional compression was established immediately. Five minutes later, the patient appeared obvious chest tightness and shortness of breath symptoms, so the compression was stopped. Then, we urgently contacted the drug bank, and were informed that the prothrombin was out of stock. Meanwhile, the artery diameter in pseudoaneurysm was larger (0.7 cm). It was reported that the artery diameter larger than 0.8 cm was the relative contraindication of prothrombin or anhydrous alcohol injection. In addition, the patients had higher requirement for the perfect appearance. After multidisciplinary consultation, we decided to use microwave ablation to seal the blood vessels in pseudoaneurysm (Fig.3), followed by removing the hematoma using UGVAB again (Fig.4). On the second day after treatment, the compression and pain symptoms of the patient were obviously relieved.

## DISCUSSION

The most common complications of UGVAB are the hematoma and infection. At present, hematoma is the main complication found in our hospital, which can be spontaneously absorbed in 3-6 weeks. The vascular injury is a rare complication of UGVAB. It has been reported that, the incidence of pseudoaneurysm is very low in iatrogenic injury.^9^ Pseudoaneurysm is easy to occur in the elderly patients with arteriosclerosis and patients who take anticoagulants.^10^,^11^ However, the patient in our study was a healthy woman. The hematoma gradually occurs after the bandage is removed at 48 hour after UGVAB. This belongs to late-onset hematoma, which is seldom reported in the literatures. The formation of pseudoaneurysm is often due to the injury of the arterial wall by iatrogenic trauma.^12^ In this injury, the local three-layer structure of arterial wall is ruptured, and the blood seeps out to the adjacent tissue, forming a fiber package. In color Doppler ultrasound, the pseudoaneurysm can be confirmed by displaying the filling and outflow of the artery lumen through a small channel at the initial vascular injury site. Because the diameter of blood vessels in the breast is usually only 2-3 mm, the occurrence of breast pseudoaneurysm is very rare. The common treatment methods of breast pseudoaneurysm include the follow-up, ultrasound-guided directional compression, prothrombin injection, surgical intravascular embolization and open surgery.^5^-^8^ In this study, we used microwave ablation to treat the breast pseudoaneurysm, followed by using UGVAB to eliminate the hematoma, and have obtained good therapeutic effect.

From this case, we obtain some experiences for breast pseudoaneurysm. Before the interventional operations including core needle biopsy and UGVAB, the systematic ultrasound examination should be performed. In the needle entering channel, the obvious blood vessels should be avoided, so as to reduce the unnecessary vascular injury. When the pseudoaneurysm occurs, the patient’s condition, pseudoaneurysm situation and hematoma size should be comprehensively considered, combined with the multidisciplinary consultation, for selecting the best treatment strategy.
